# Professional dancers’ beliefs and conceptualisations of their posture and movement: A qualitative research study

**DOI:** 10.1371/journal.pone.0339568

**Published:** 2026-02-09

**Authors:** Andi Guo, Xiao Zhang, Marshall Hagins, Marijeanne Liederbach, Paul W. Hodges

**Affiliations:** 1 Centre for Innovation in Pain and Health Research, School of Health and Rehabilitation Sciences, The University of Queensland, Brisbane, Queensland, Australia; 2 Department of Physical Therapy, Long Island University, New York City, New York, United States of America; 3 Harkness Centre for Dance Injuries, NYU Langone Orthopedic Hospital, New York City, New York, United States of America; Università degli Studi di Milano: Universita degli Studi di Milano, ITALY

## Abstract

Many issues are unresolved regarding posture and movement and their utility as target for rehabilitation and function. As movement experts, elite ballet and contemporary dancers could provide insights regarding whether some postures/movements are ideal, if and how they can be changed, and valuable insight into the apparent paradox between their “exceptional” posture/movement quality and high incidence of musculoskeletal injury and pain. This study aimed to understand dancers’ beliefs and conceptualizations about posture and movement, consider how this relates to theories of motor control and learning, and whether this has relevance for rehabilitation and injury prevention in non-dancers. This study implemented an inductive qualitative design. Thirty-six participants (12 males, 24 females) with 6.9 ± 4.3 years of experience as professional dancers (ballet or contemporary dance) participated. Data were collected via semi-structured focus group interviews. Thematic analysis used an inductive approach to categorise and structure data into codes, themes and sub-themes. Themes were refined and finalised after multiple team discussions. Four themes emerged: (i) Posture being conscious or subconscious, (ii) Posture in “dancer mode” and “non-dancer mode”, (iii) Posture as an ideal, (iv) Adjustment and improvement of posture. Each theme was summarised by a series of sub-themes. Dancers have a detailed and multifaceted understanding of posture and movement. They reconcile the contradiction between good posture and movement and injury by the extreme demands required to achieve aesthetics of dance. Some of their perspectives have potential relevance in injury prevention and rehabilitation in the general population.

## 1. Introduction

Human posture and movement serve diverse purposes. They enable interactions between the body and its surroundings, they are used to communicate intentions and attitudes [1], and are argued to be relevant for pain and injury [2]. Posture and postural control are dynamic concepts. Posture refers to the *alignment* of body relative to the gravity/environment and alignment of body segments relative to each other [3]. Postural control refers to the sensory (afferent input providing information of body position, movement orientation relative to the environment) and motor processes (spinal and supraspinal control mechanisms, muscle activation) involved in the *maintenance* of equilibrium (maintenance of centre of mass over the base of support) and orientation of segments relative to each other and includes an essential contribution of movement [4]. Multiple strategies are used to maintain postural control. These include anticipatory or feedforward strategies when the challenge can be anticipated, feedback mediated control when the challenge is not predictable, and tonic strategies of ongoing muscle activity to provide an ongoing stiffness to segments [4]. Although there is considerable discussion that some postures are more ideal than others, most research suggests a diversity of individual variation [3]. Regardless, postural correction is a common component of rehabilitation for pain and function [5]. There are many unresolved issues regarding posture and movement and their utility as a target for training for healthy function as well for the prevention and treatment of pain. For instance – is there an ideal posture [3], can posture be consciously controlled or trained [6], and does change in posture require ongoing practice?

Elite dancers (e.g., professional and pre-professional), including ballet and contemporary dance, are a unique group who require mastery of a specific repertoire of postures and movements to entertain, tell stories and convey emotion, among other purposes. It is broadly accepted that elite dancers differ from non-dancers in their posture and postural control capabilities [7,8]. For instance, elite dancers have different spinal posture (e.g., less thoracic kyphosis, lumbar lordosis and anterior pelvic tilt in standing), greater spine flexibility and more extensible hamstring muscles [9]. Dancers exhibit enhanced neuromuscular responses and proprioceptive acuity, which is linked with superior postural control and balance when dealing with postural instability and perturbations [7,8,10]. Understanding how elite dancers conceptualise posture and movement is likely to provide meaningful insights regarding whether some postures and movements are ideal, and if and how it can be changed.

Dancer’s conceptualisation of posture and movement might also guide understanding the relationship between posture/movement and pain. Despite their superior postural control, elite dancers have a high incidence of injuries. Up to 80% of ballet dancers and 89% of contemporary dancers experience musculoskeletal injuries across their career [11,12]. This apparent paradox is worthy of exploration. Recent research has ignited debate regarding the role of posture and movement in prevention and rehabilitation of musculoskeletal conditions [2,13]. Some systematic reviews highlight an unclear relationship between posture in sitting and clinically relevant episodes of low back pain, and evidence for the relationship between posture and low back pain during weight-lifting activities comes from small studies with poor methodological quality and inadequately validated measures of posture [14,15]. Although a firmly held clinical belief is that certain postures and movements can be risk factors for development of pain and injury, the current debate questions this assumption and suggest their importance for injury may have been overstated. The apparent contradiction between the “exceptional” posture and movement quality of ballet and contemporary dancers [16], and their high incidence of musculoskeletal injury and pain could provide important insight. It is possible that some postures and movements are ideal, but despite their “ideal” nature they are associated with pain an injury because of the high demand of training and performance (as has been suggested [17,18]) and the use of postures and movements outside typical biomechanical parameters. Alternatively, the paradox might also infer that postures and movements that are generally perceived as “good” or ideal across contexts are not actually relevant for prevention of pain and injury.

Understanding how dancers conceptualise posture and movement could help address several key issues. These include: how dancers perceive the role of posture and movement in injury – in view of their regular experience of pain and injury; the contentious concept of an “ideal” posture and movement strategy [19]; what features they considered characterises good quality posture and movement in the context of dance and non-dance activities; whether the unique nature of posture and movement in dancers is innate, trained or both; and whether dancers need to continue to “practice” or reinforce posture and movement for it to be maintained. The primary aim of our study was to understand dancers’ conceptualization of posture and movement to address these issues. A secondary aim was to consider how these conceptualisations relate to theories of motor control and learning, and the possible implications that these dancer perspectives might have for understanding posture and movement in the general population, rehabilitation and injury prevention.

## 2. Method

### 2.1. Study design

This study used a qualitative design based on in-depth interviews to understand dancers’ perspectives. Some dancers with current limitation to dance because of pain were included. Most or all dancers will experience pain and injury at some point, and we acknowledge that this will shape their perspective of the relationship between posture and movement. Further, we included ballet and contemporary dancers. We considered these two issues to be critical to ensure diverse perspectives were captured. We used semi-structured group interviews to collect data about professional dancers’ beliefs and conceptualisation of posture and movement. Themes from narratives provided by participants were derived via thematic analysis using an inductive approach.

### 2.2. Participant selection

Thirty-six individuals who self-identified as elite ballet or contemporary dancers were recruited via social media, word-of-mouth, dance community notice boards and through the networks of professional dancers in New York City. Recruited participants were encouraged to invite colleagues to consider participation. Selective sampling was used to ensure a range of ages, dancing styles (both ballet or contemporary, and from different companies) and history of musculoskeletal pain (e.g., current and previous pain, different regions of the body) were represented. To be included, participants had to be professional ballet or contemporary dancers earning a primary wage as a dancer or full-time students in a university setting with dance as their current major, and 18 years of age or older. The study was approved by the institutional Medical Research Ethics Committee (The University of Queensland: 2019001451) and conducted in July 2019. Participants provided written informed consent.

### 2.3. Procedure of data collection

Focus group discussion has been shown to be useful for gathering information from people with similar backgrounds to discuss a specific topic of interest and was used here [20]. Five face-to-face semi-structured focus groups were directed by three researchers (PH, ML and MH) and each was conducted with a different group of seven or eight dancers. The topics for discussion were developed by PH, ML and MH and involved informal discussion with a group of dancers and dancer teachers prior to the formal focus groups. The discussion during the focus groups followed an interview outline (see Supplement S1 File for semi-structured focus group guide). PH led the focus groups. He has experience in leading focus groups and qualitative research but has limited experience in dance which enabled him to remain impartial regarding the main purpose of this study. During the focus group, with input from ML and MH, PH proposed issues for discussion according to the interview outline, and if necessary, clarified meaning and probed more deeply. Due to the exploratory nature of this study free-flowing conversations were allowed to enrich the depth and breadth of the data. The script was piloted with a group of 5 dancers 12 months prior to the focus groups. During these discussions, dancers were asked to reflect on issues such as ‘when’, ‘how’ and ‘why’ they think about their posture and movement.

Each focus group discussion lasted for approximately one hour and was audio-recorded. Saturation, determined through iterative data analysis during the period of data collection, was reached when repetitive information with sufficient richness and variability related to study aims could be found. Demographic data regarding age, gender, dance experience and age were collected via a paper form provided to the participants on arrival at the focus group.

### 2.4. Theoretical underpinning

Thematic analysis as described by Braun and Clarke [21] was conducted to generate understanding of dancers’ views and experiences on posture control from broad perspectives. An inductive approach was applied to minimise the influence of pre-existing scientific exploration and our own theoretical interpretation of results. This procedure first identified codes by selecting elements of the raw data that conveyed meaningful information. Themes and sub-themes emerged from these codes as broader categories that represent patterned response.

### 2.5. Data analysis

The research team consisted of two final year physiotherapy students (AG and XZ) and three senior academics (PH, MH and ML). MH and ML are experienced physiotherapists and researchers who have worked with professional dancers and dance-related injuries for over 25 years. MH is a former professional dancer. PH is an expert in movement control, pain and rehabilitation. The recorded interviews were transcribed verbatim using Otter software (AISense, Mountain View, USA). Two investigators (AG and XZ) examined the transcribed interviews to correct any errors from the automatic transcriptions. Participants were de-identified during this process. As AG and XZ familiarised themselves with the data, they identified and sorted significant parts of dancers’ opinions into codes and then categorised them into themes using data management software (Nvivo version 12, Lumivero, Denver USA). The relevance of the themes was considered for the purpose of this study and only themes related to dancers’ conceptualisations and beliefs of posture and movement were extracted. PH independently read the transcripts and also identified relevant themes. AG, XZ and PH met to identify themes, to discuss accuracy of theme identification, and to refine the terminology used to describe them. Four major themes were identified. AG and PH developed graphical representation of the themes to consider subthemes, and to consider the connections within and between the themes. The graphical representation of the initial themes were presented to MH and ML as the starting point for a series of four on-line discussions to gain their insight within the contexts of their expertise in dance-related research and clinical experience. These discussions resulted in modification and refinement of the themes. Consolidated Criteria for Reporting Qualitative Studies (COREQ) questionnaire was used to ensure rigour in reporting results [22].

## 3. Results

**Fig 1:**
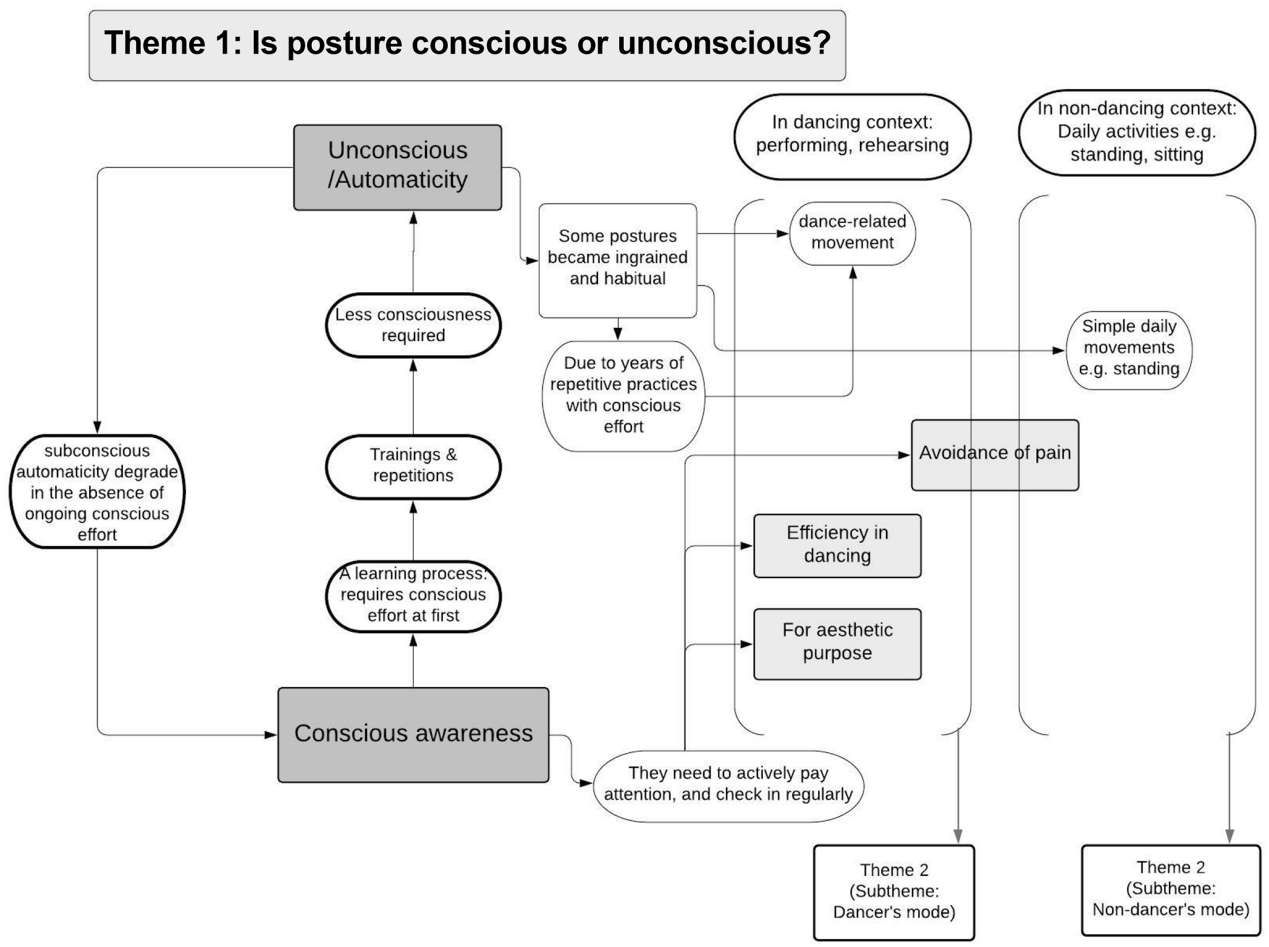
Theme 1: Posture being conscious or subconscious. Concepts and subthemes along with interconnections with other themes.

**Fig 2:**
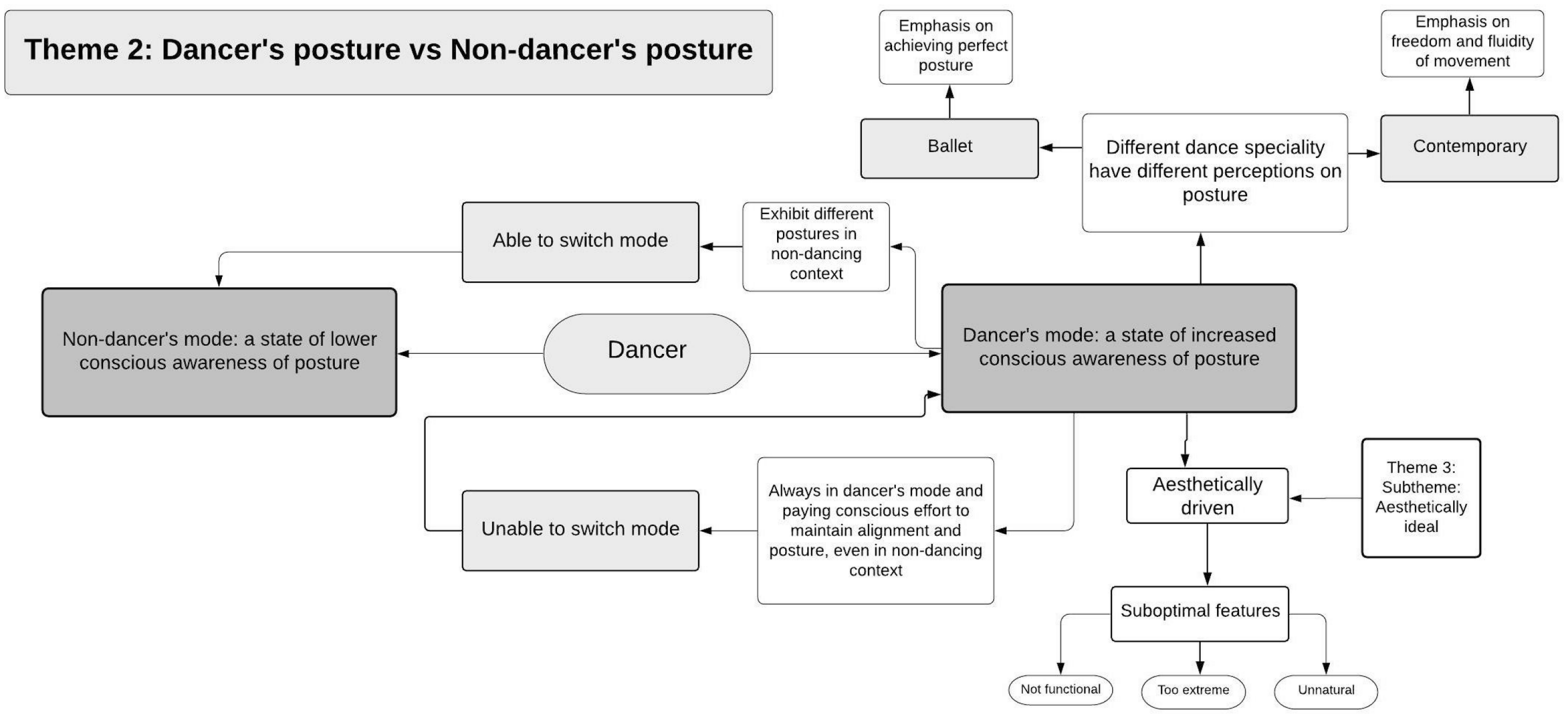
Theme 2: Posture in dancer mode and non-dancer mode. Concepts and subthemes along with interconnections with other themes.

**Fig 3:**
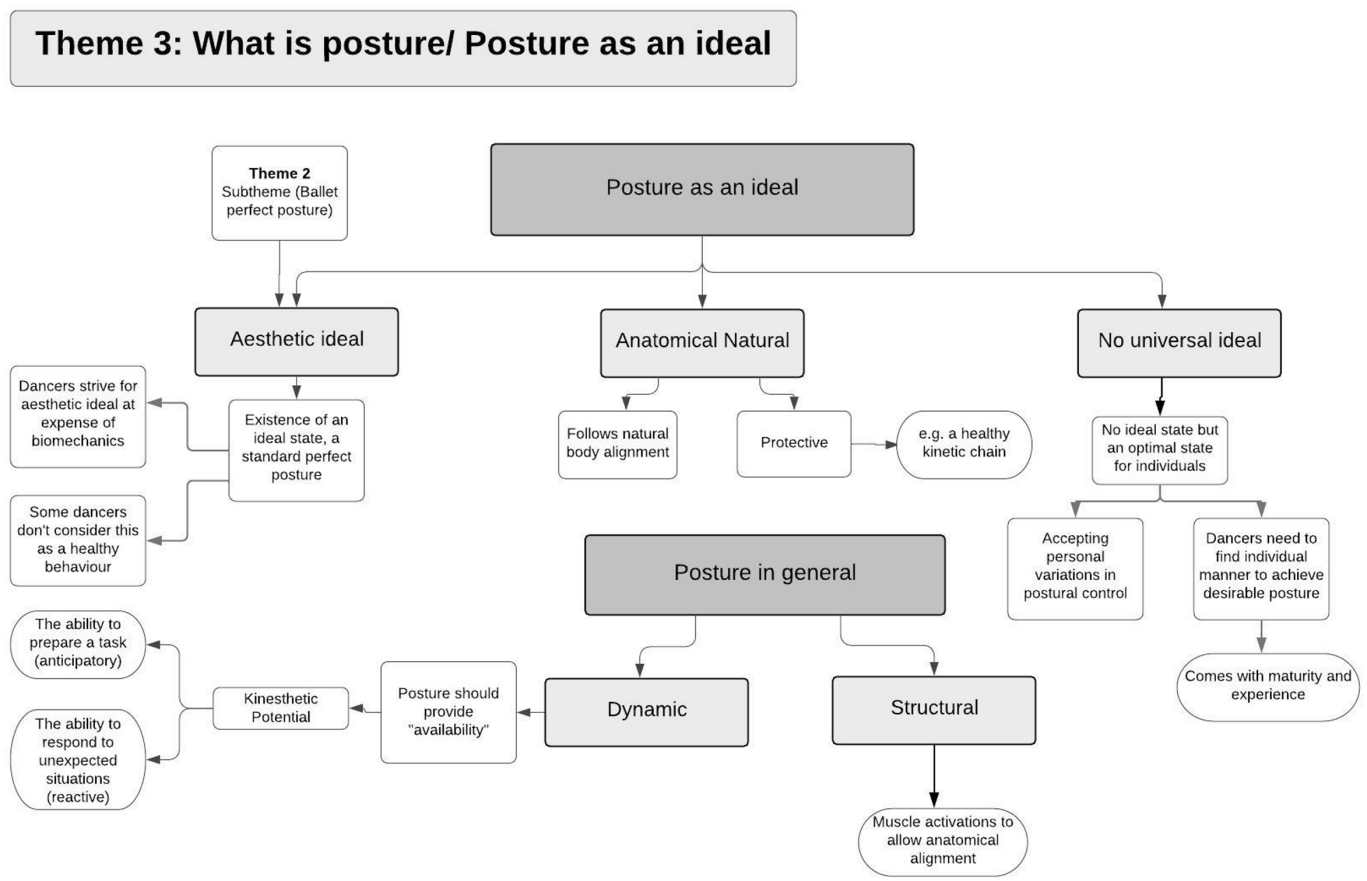
Theme 3: Posture as an ideal. Concepts and subthemes along with interconnections with other themes.

**Fig 4:**
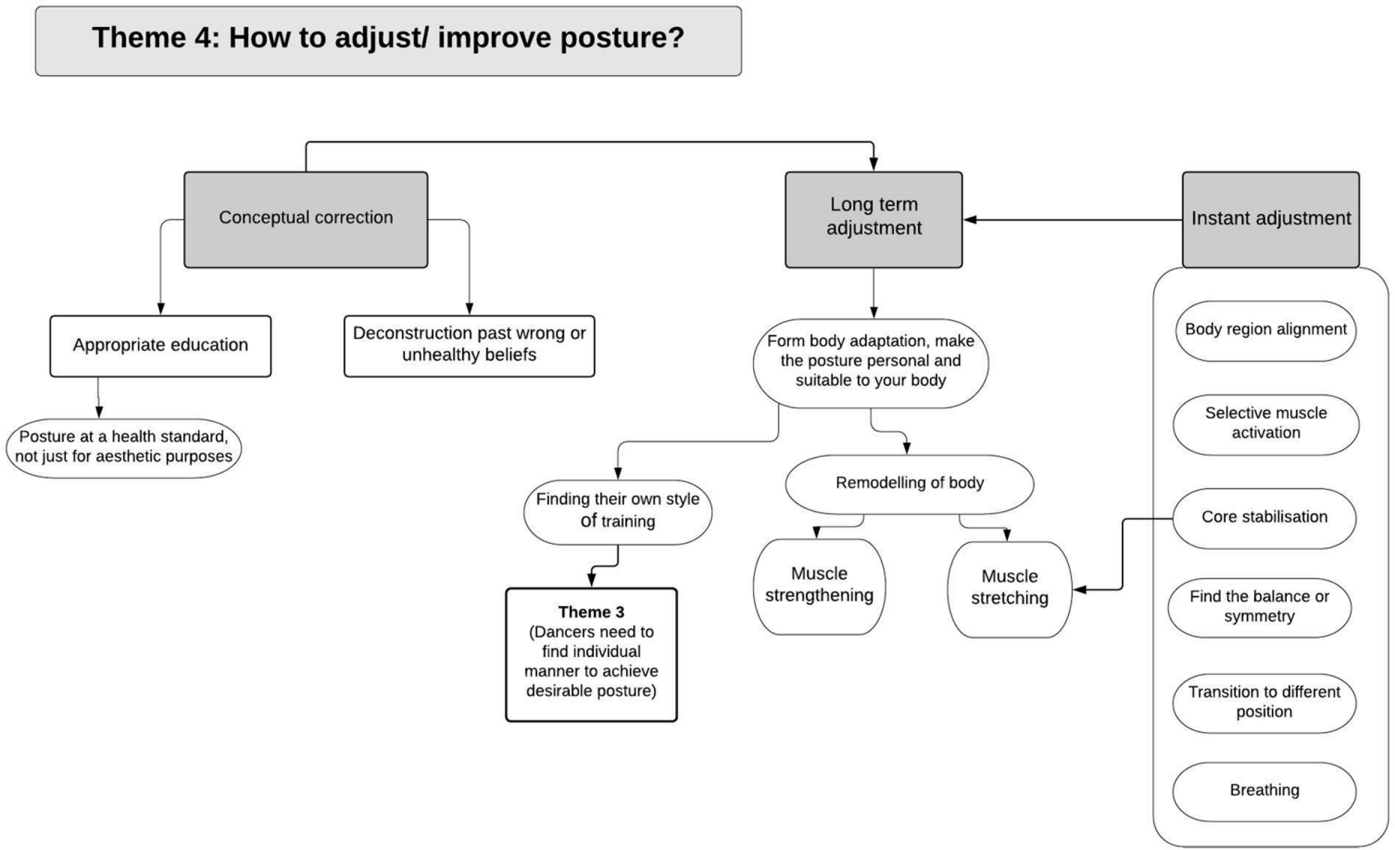
Theme 4: Adjustment and improvement of posture. Concepts and subthemes along with interconnections with other themes.

Demographics and dance experience of the included dancers are presented in Table 1. Analysis of the focus group data revealed four main themes that encompassed dancers’ perceptions of posture and movement: (i) Posture being conscious or subconscious, (ii) Posture in “dancer mode” and “non-dancer mode”, (iii) Posture as an ideal, and (iv) Adjustment and improvement of posture. Each theme could be summarised by a series of sub-themes. Figs 1–4 present an overview of the themes, concepts and the interconnections between them. Supplement S2 File provides a high-resolution version of all themes and the interconnection between them. The following summary describes the themes and sub-themes including supportive quotes from the participants which are presented in Table 2 using reference to the theme number (T) and quote number (Q) using the following format - T1Q1, T1Q2, T2Q1, and so on.

**Table 1:** Demographic characteristics of study participants.

Participants	
Age (years)	
Mean ± SD (range)	27.9 ± 5.2 (19-43)
Gender	
Female	66.7%
Male	33.3%
Dancing style	
Ballet	8
Contemporary	28
Formal dancing trainings (Years)	
Mean ± SD (range)	16.1 ± 6.4 (6-27)
Professional dancing (Years)	
Mean ± SD (range)	6.9 ± 4.3 (1-17)
Dance Load (Hours/week)	
Mean ± SD (range)	30 ± 12.5 (7.5-50)
Current full dance and rehearsal schedule	
Yes	26.7%
No	74.3%
Pain that has limited dance in past 2 months	
Yes	37.1%
No	62.9%
**Pain location (ever)** (N (percent))	35 (97%)
Low back (N (percent))	27 (75%)
Neck/upper back (N (percent))	16 (44%)
Shoulder (N (percent))	18 (50%)
Hip (N (percent))	18 (50%)
Knee (N (percent))	23 (64%)
Ankle (N (percent))	21 (58%)
Other (N (percent))	10 (28%)
None (N (percent))	1 (3%)
**Pain location (current)** (N (percent))	19 (53%)
Low back (N, pain intensity mean (range)	2, 3.5 (2-5)
Neck/upper back (N, pain intensity mean (range)	2, 2.5 (2-3)
Shoulder (N, pain intensity mean (range)	3, 2.7 (2-3)
Hip (N, pain intensity mean (range)	2, 4.5 (3-6)
Knee (N, pain intensity mean (range)	5, 5.4 (2-8)
Ankle (N, pain intensity mean (range)	5, 2.8 (2-4_

**Table 2:** Primary themes and categories from dancers’ beliefs and conceptualisation on posture and movement, with representative quotes.

**Theme 1: Posture being conscious or subconscious**			
**Posture is controlled by conscious awareness**	**Avoidance of pain**	*“I do actively think of my posture, where my public alignment is, how I’m holding my, my shoulder is if I have a backpack on where I’m placing, stacking my bones as I physically climb the stairs to limit the pain or discomfort I might experience on my knees.”*	*T1Q1*
	**Improvement of efficiency in dancing**	*“When I’m dancing I’m constantly checking in. Just because like, generally when I am unable to achieve a step it’s generally due to alignment. And so if I go back to center, realign, I’m able to execute it.”*	*TQ2*
	**Aesthetic purposes**	*“So then I started thinking more and more of it, trying to get back the aesthetically good posture, ballet standard posture.”*	*TQ3*
**Posture can be subconscious**	**Training effect**	*“That it doesn’t feel like I am doing it on purpose to your point of our body knows what to do. It’s almost like it is a subconscious unconscious thing that just occurs because of the training. “*	*T1Q4*
	**Translation to daily activities**	*“But it’s just sort of it’s becoming ingrained to us to have our shoulders back our chest be sort of up, be standing straighter than necessary”*	*T1Q5*
**Level of consciousness can alter and it is a reversible process**	**Translation of conscious to subconscious, after practices**	*“I know that I tend to climb stairs with my quads, rather than using my glutes. And so just making sure to, like, consciously do it. Now that I’ve sort of gotten more into the habit of it. And to think about it less, I can just check and be like, Oh, yeah, I’m doing it, as opposed to each step thinking about it.”*	*T1Q6*
	**Loss of subconscious control**	*“So the ideal that you try to achieve doesn’t necessarily become automatic, because if you don’t practice it, you lose some of it.”*	*T1Q7*
**Theme 2: Dancer’s posture vs Non-dancer’s posture**			
**Posture in dancer’s mode:**	*“When I’m in the dance environment I’m constantly aware of, you know, am I placing my feet, my knee tracking right over my feet? Is my spine aligned?”*		*T2Q1*
	**Suboptimal features at expense of biomechanics**	**Too extreme: “** *There’s that thing that happens in dance in general, where you can push too far from one and from, from one suggestion of what’s perfect, way over to an extreme right and then puts sort of weird habits on the bone structure.”*	*T2Q2*
		**Not functional:** “*Yes, but those are dance, that’s not functional. We are asked to do crazy. It’s all for, and what is it called? No and like aesthetics, it’s all for aesthetics, you want to look cool.”*	*T2Q3*
		**Unnatural:** *“It’s not a natural way for our bodies to be enjoying it. The way we’re saying it the way we do this. It’s meant to be, you know, it’s not human, it’s like, really like force, like turning out.”*	*T2Q4*
	**Dance style specificity**	**Contemporary** *: “But in contemporary dance, because it’s more movement based, it’s more fluid that allows you to figure out how your body reacts to the movement. And like how you make it your own and how it works for you in the sense.”*	*T2Q5*
		**Ballet:** *“But I’m gonna speak to ballet, like there is an ideal. And generally one of twenty dancers on stage where you all feel the same.”*	*T2Q6*
**Posture in non-dancer’s mode**	*“In my daily life, the only time I, I mean I try to prepare myself, but I don’t really think about as much”*		*T2Q7*
	**Unable to switch mode**	*“So it’s, it’s sort of, I think, my, my thinking has shifted to needing to think of, think about my posture when I’m outside of dance, so that so that I don’t develop bad habits that would impact dancing.”*	*T2Q8*
	**Able to switch**	*“Yeah, I don’t think that I’m actively aware of stacking those bones anymore. Just kind of switch into that mode.”*	*T2Q9*
**Theme 3: What is posture/posture as an ideal**			
**Posture is structural:**	*“I think once we all figure out how our bodies anatomically align correctly. Then comes the posture.”*		*T3Q1*
	*“Posture being more superficial thing where, where the skeleton sort of sits and rests, and muscles and ligaments affect where the bones sit.”*		*T3Q2*
**Posture is dynamic**	**Availability/ Kinaesthetic potential**	**To prepare:** *“Like enabling yourself to organize your body in such a way that you can achieve these tasks, optimising our bodies for these things, preparing all this stuff.”*	*T3Q3*
		**To respond:** *“So the posture has to be this thing, that readily available, has availability to shift with a quick so it doesn’t crack because it’s brittle.”*	*T3Q4*
		**Pendulum and baseline:** *“It’s kind of the base. And I can move from that base in either direction, not spatially direction forward or backwards. But it’s like a pendulum, like posture is where there’s the downswing, the ease in between can go to either spectrum from that neutral posture.”*	*T3Q5*
**Posture as an ideal**	**Generalised Ideal**	*“So then I started thinking more and more of it, trying to get back the aesthetically good posture, ballet standard posture.”*	*T3Q6*
		**“***Perfect posture is not natural***”**.	*T3Q7*
		*“And then you take that on as perfect posture. So then you leave the room and attempt to hold yourself that we constantly which can become actually unhealthy.”*	*T3Q8*
	**A natural, safe, and healthy state is ideal:**	**Natural** *: “And like where it naturally wants to be plugged in. It’s more, it’s like a neutral, grounded place that the bone is, has evolved to be in a human body.”*	*T3Q9*
		**Healthy** *: “What is good just to be functioning at a normal, healthy kinetic chain.”*	*T3Q10*
	**No ideal state**	**“** *Just observing how different postures serve different purposes, and realising that there isn’t one posture that is an ideal”*	*T3Q11*
		**Individualised optimism:** *“I think it’s like, perfect versus optimal. I feel like not going towards the perfect, but going towards the optimal for you. “*	*T3Q12*
		**Realisation comes with maturity and experience**: “*And I think all of us probably went through a phase where we are also trying to get that and you get older, and then you gain a lot of information, you do other dance styles, and then you start to learn on your own how to take on a more intelligent way to move. And I think a lot of that just comes with like maturity of learning your body, and maybe not so much like, you know, harmful training, sometimes it can do that. But sometimes it’s just, you just need the time to realize it.”*	*T3Q13*
**Theme 4: How to adjust or improve posture**			
**Correction through instant adjustment:**	**Anatomical alignment**	*“I like to think about all the points I can make where, where do they fall on my body”.*	*T4Q1*
	**Muscle activation**	*“I try to aspire stronger activation of the right hamstring, and stronger glutes. And so I can properly create, I could properly activate my hamstring to lengthen so I can put myself in a neutral position to lengthen through my lower lumbar back*.”	*T4Q2*
	**Change position**	*“I’ve been in this same position for a while, I should like shift or I’m always like moving on even in this chair like notice I’m just like rocking back and forth.”*	*T4Q3*
	**Core engagement**	*“How am I using my body to ensure I’m not hurting myself, so that idea of like, I’m going to lift something heavy, so and engage my core lift from the legs.”*	*T4Q4*
	**Breathing**	*“And breathing helps integrate all of that stuff, right? It helps access my blood flow and helps your muscles move.”*	*T4Q5*
**Correction over long term**	*“And I think this part takes time, internally, like strengthen deep muscles that support that structure, and make it more personal.” Another response is to train in their own style. what’s helped me support that is actually my movement training, whether Pilates, weightlifting, or, that’s helped me. But if I just kept going one cell to the next, I’m like getting lost in translation, my body just gets boxed up too much sugar gets too much there, I need to have my own practice in between to support myself.”*		*T4Q6*
	*“So I feel like even across the other side of the dance, you have to adjust your posture, sort of like deconstruct what you think posture is.”*		*T4Q7*
**Conceptual correction**	*“I think that generally in the dance world, I think, in education there, there can be more, you know, with anatomy and lessons and while there while the dancers are still young.”*		*T4Q8*

### 3.1. Theme 1: Posture being conscious or subconscious

Participants acknowledged that posture during dance and non-dance was controlled both consciously and subconsciously (Fig 1).

#### 3.1.1. Subtheme: Posture is controlled by conscious awareness and intent.

Some dancers believed conscious awareness is required to maintain and modify their posture. They reported a need to actively pay attention and “check in” on their posture regularly throughout the day in both dancing and non-dancing contexts. Dancers explained that such consciousness of posture is motivated by their aim to avoid pain in the immediate or distant future (T1Q1), improve efficiency in dancing (T1Q2), and for aesthetic purposes (T1Q3).

#### 3.1.2. Subtheme: Posture can become subconscious and autonomous.

Rather than controlling postures consciously all the time, dancers stated an unconscious automaticity could be developed which requires less conscious awareness (T1Q6). Some explained that the automaticity required repetitive practice with conscious effort and was reinforced over time. These trained postures were primarily held or present in the context of dance (T1Q4). Some dancers reported that these trained postural habits could transfer to some non-dancing tasks or daily activities such as standing and walking (T1Q5).

#### 3.1.3. Subtheme: Level of consciousness can alter, and it is a reversible process.

Some dancers considered that subconscious automaticity of control of specific postures diminishes if it is not reinforced by ongoing conscious effort to practice “good” posture. They suggest that reliance on subconscious control is insufficient to maintain good posture (T1Q7) and that posture can degrade and deviate from its desirable form due to lack of practice.

### 3.2. Theme 2: Posture in dancer mode and non-dancer mode

As dancers reflected upon their postures, they conceptualised two different contexts for posture – “dancer mode” and “non-dancer mode”, which alters in its form based on contextual demand (Fig 2).

#### 3.2.1. Subtheme: Posture in dancer mode.

“Dancer mode” was described as a state of increased conscious awareness of posture focusing on the specific aesthetic demands of the dance style of the dancer (T2Q1). Some claimed that certain postures in dancer mode produced suboptimal impacts for body because of the pursuit of aesthetics at the expense of biomechanics, which would ultimately impact their dance performance and health. Potentially problematic features were described as being too extreme (T2Q2), not functional (T2Q3) and unnatural (T2Q4). Dancer mode was understood within the context of each dancer’s philosophy, their dancing background and genre. A mutually agreed statement was that contemporary dance tends to emphasize freedom and fluidity of movement (T2Q5), whereas ballet tends to emphasize achievement of a specific “perfect” form (T2Q6).

#### 3.2.2. Subtheme: Posture in non-dancer mode.

“Non-dancer mode” was described as a state of lower conscious awareness of posture than dancer mode (T2Q7). Dancers switch between these two “modes” based primarily on contextual demands. Some dancers reported that regardless of the context, they are always in “dancer mode” meaning that they conducted non-dance activities in dance-related posture with frequent conscious effort to maintain a specific postural alignment or movement characteristic (T2Q8). Others reported finding it easy to switch to non-dancer mode with distinct repertoire of postures that did not involve conscious attention (T2Q9).

### 3.3. Theme 3: Posture as an ideal

This theme considers the conceptualisation of posture in general and posture as an ideal state (Fig 3).

#### 3.3.1. Subtheme: Posture is structural.

Many emphasised that anatomical alignment is the foundation of posture (T3Q1). Based on their anatomical knowledge, some specified that alignment is about the skeletal alignment and muscle control (T3Q2).

#### 3.3.2. Subtheme: Posture is dynamic.

Dancers stated that posture should be dynamic rather than static alignment. They emphasised that posture should provide “availability” of potential motion. One participant described this using the term “kinaesthetic potential”, specifying that the dynamic component of posture should provide dancers the capability to prepare for a task (T3Q3) and to respond safely to unexpected situations (T3Q4). One participant described the dynamicity using a model of pendulum (T3Q5), reinforcing posture should allow wide ranges of movement towards different directions.

#### 3.3.3. Subtheme: Posture as an ideal.

Various opinions were revealed on the topic of ideal posture.

***Aesthetic Ideal*:** Some dancers proposed that posture has an ideal state. As performing artists, some dancer’s ideal posture is based on the aesthetic requirements. One dancer reflected that the way they improved their posture was by resetting themselves back to “aesthetically good posture, ballet standard posture” (T3Q6). However, some dancers did not consider this as healthy or natural behaviour and embedded the concept of “unnatural perfection” (T3Q7) within their responses. Many stated that maintaining these standardised postures in the long-term could lead to an extreme anatomical alignment (T3Q8) and acknowledged a potential long-term cost.

***A natural, safe, and healthy state is ideal*:** Dancers emphasised that posture should be held in a “neutral, grounded” way (T3Q9) and be “functional at a healthy kinetic chain” level (T3Q10). This contrasts with the concept of unnatural idealism.

***There is no single ideal state but different unique optimal states for individuals*:** In contrast to the preceding subthemes that relate to the attitudes related to the specific genres, some participants expressed a different, perhaps individual, perspective that suggested that there is no ideal state for posture (T3Q11). One participant revealed that although there is no perfect posture, one can still find what is optimal for themselves. They emphasised the concept that posture is highly individualised and the importance of accepting personal variations in posture (T3Q12). There was a realisation that it was necessary for each dancer to find their own individual manner to achieve the perceived optimum, and that the success of this could be linked with experience and maturity in their dancing career (T3Q13).

### 3.4. Theme 4: Adjustment and improvement of posture

This theme focused on how dancers adjust or “improve” their postures and three non-mutually exclusive subthemes were observed and discussed (Fig 4).

#### 3.4.1. Subtheme: Correction through instant adjustment.

When dancers had detected that their posture became “wrong or uncomfortable”, they would instantly respond and ‘correct’. Common responses included change in body region alignment (T4Q1), alteration in muscle activation (T4Q2), performance of a “core stabilisation” manoeuvre (T4Q4), returning to balanced or symmetrical status, transition to another position (T4Q3) and breathing techniques (T4Q5).

#### 3.4.2. Subtheme: Correction over long term.

Some participants indicated that long-term training was needed to change postures they considered to be suboptimal. This time was considered necessary to develop body adaptations such that the posture was suitable for their own bodies. Some dancers advised strategies including muscle strengthening and muscle lengthening (T4Q6). Some also learnt from experience with efforts to consciously deconstruct learned dance postures to achieve a new form or dance genre or specific functional task (T4Q7).

#### 3.4.3. Subtheme: Conceptual correction.

Many participants described that a change in conceptualisation and better understanding of posture would be beneficial for their dancing career. Several participants had complaints about dancers having a narrow education which only allowed them to understand posture from an aesthetic perspective but not based on health standards (T4Q8).

## 4. Discussion

This study provides insight into posture and movement from the perspective of elite dancers who are considered experts in postural control. We identified several major conceptualisations that dancers have regarding posture and movement of relevance to the understanding of ideal posture, its potential to be modified, and the apparent contradiction between posture/movement and pain/injury. Dancers believed that conscious effort is required to refine posture and required for maintenance over time. They speculated that specific postures help to improve their quality of movement, prevent injuries and pain, and achieve the aesthetics required by dance, but that these outcomes might not be mutually consistent with some relationship between attainment of aesthetics and injury. Notably, dancers reinforced that posture is dynamic, adaptable and individualised.

### 4.1. Postural control is a continuous motor learning process

Dancers reinforced that when learning a novel or unfamiliar movement and posture, the level of attention transitions from conscious to subconscious after repetitive task practice. This concurs with the widely accepted models of motor learning [23], which consider three progressive stages: cognitive, associative, and autonomous, with reduced attention as motor learning progresses [24]. Although it might be expected that professional dancers would perform at the autonomous level with low attentional demand, they acknowledged that despite repetitive practice, absence of ongoing conscious attention over time can “degrade” the quality of posture from its desirable form. Dancers suggested that the permanence of motor skill acquisition cannot be assumed, and high-level refinement required continuous conscious effort for maintenance.

That dancers describe attention to specific postures and movements (internal focus of attention) that might appear to contradict the concept that movement performance is better when attention is directed towards the external effects of an action (external focus of attention) [25]. There are two issues to consider. First, dancers implied that this was not considered in every task but described a concept of “checking in” periodically. Second, continued engagement of cognitive awareness to fine-tune movement concurs with observations of Ericsson [19] that suggest learning differs between skills needed for everyday tasks and those needed for expert performance. For everyday activities the goal is to learn a movement with satisfactory quality in minimum time and become autonomous. In contrast, expert performance involves increasingly complex mental representations to attain higher levels of control of performance that remain within the cognitive and associative phases [26]. Dancers also identified greater conscious awareness in “dancer” than “non-dancer” mode to improve the quality and efficiency of dancing. These findings suggest dancers acknowledge the necessity to maintain a constant high degree of conscious awareness to continuously optimise and refine the precision of posture, despite their advanced expertise in movement.

### 4.2. Posture and movement are highly individualised

Dance is generally considered to involve achievement of specific idealised dance postures, yet dancers argue that individual variation is required to achieve this goal. This is not unique to dancers. Recent data show individual differences in muscle activation patterns in gait, despite similar performance of the task [27]. Of note, dancers argued that a feature of maturity as a dancer was the realisation that they can, and should, find their own optimal solution to match their own biomechanics, kinematics and physiology and not merely duplicate others to achieve the dance aesthetics. Some examples have been researched. Individual differences in shape of the acetabulum, and neck of femur can limit the range of hip external rotation and requires compensation by other segments, such as greater lumbar lordosis, external rotation of the knee, or valgus heel with forefoot pronation [28]. Although these compensations might enable the position required by dance, this may place additional stress on segments that compensate. Absence of compensation is also likely to be problematic; dancers who lack sufficient ankle plantarflexion for *pointe* or *demi-pointe* positions, will often attempt to force plantarflexion and place greater stress on the posterior and lateral ankle structures, and predisposes themselves to varus malalignment at the foot-ankle complex [29]. Consideration of individual variation and whether to compensate or train requires consideration.

Dancers also indicated that achievement of a desired posture depends on elements of fitness beyond the specific muscles and joints involved in the task, such as physical capacity and “core stability”, which refers to training of trunk muscles to maintain control of the proximal regions of the body, particularly the spine, during limb movements. Consistent with this proposal are data that show improvement in ballet dancers’ alignment of *demi-plié* and *passé* dance positions after participation in a summer intensive dance program that trained lower abdominal muscle strength [30]. This concept reinforces that optimum state of posture requires attention to features beyond those of each individual segment.

### 4.3. The dynamicity and adaptability of posture

Dancers emphasised the static and dynamic nature of posture. A common concept was that posture is inherently dynamic and can never be static. In quasi-static situations, such as standing, dancers reinforced that maintenance of alignment relies on constant contribution of muscle control. This principle is congruent with evidence that even in quiet stance, small motions of spine and pelvis are required to counteract the disturbance to postural equilibrium, including those caused by breathing [31].

Dancers used the term “kinaesthetic potential” to describe the concept that maintenance of alignment in a dynamic manner enables availability of adjustments during motion such that if perturbations during movements can easily be corrected. This concurs with the view that describes stability of a dynamic system as “the ability to maintain the desired trajectory despite kinetic, kinematic, or control disturbances.” [32]. This dynamic control is particularly important for dancers who move through large or perhaps “unnatural” trajectories.

Postural control depends on the environment, intended goals, biomechanical constraints, and sensory conditions [33] and must be adaptable to changes in these elements. Some dancers indicated that components of dance-related posture and movement are preserved in everyday activities, either consciously or subconsciously, with positive and negative consequences for health. Although some data suggest gait patterns used by dancers generate greater strain on lateral ankle structures [34], other work shows that when dancers transfer their landing technique to non-ballet-specific tasks this is associated with a low rate of ACL injuries [35]. Whether this translation between contexts is beneficial or harmful has yet to be clarified.

### 4.4. Relationship between posture/movement, injury and pain

Dancers expressed a strong belief that “poor” posture places them at risk of pain and injury, and that “good” postures can prevent injury and improve efficiency of dancing. A common perspective was that sometimes injury and pain were induced by incongruency between the dance aesthetic and musculoskeletal health. That is, that postures and movements that they considered to be good/ideal in non-dance contexts might protect them from injury, but with the caveat that some aspects of posture and movement that were required for dance placed them at risk because they were too “extreme” and “unnatural”. Findings from several studies support this view: systematic review of evidence suggests specific spinal alignment increases risk for injury in professional ballet dancers [28,36]; hyperflexibility of hip joint and extreme hip motions increase susceptibility for hip impingement and instability [37]; and gait patterns commonly used by dancers induce greater medial shear force in pre-swing gait phases than that of non-dancers [34].

It is difficult to extrapolate studies that question the protective nature of posture and movement [14,15]. to dancers. The relationship needs to be considered in the context of demands and cummulative load of professional dance. For example, pain and injury may be related to demand, but outcomes might be worse if posture and movement were less ideal. Additionally, it is possible that the relationship is more complex than a focus on a single negative aspect of posture (as is often assumed in previous research). A relationship to injury might be more likely in professional dancers because of the substantial stress on the body as a result of the demands of dance, for example, the load placed on the lumbar spine to achieve greater turnout during routine movements and positions [28] and demanding training loads [36]. Regardless of the evidence, prevention of injury is a primary motivator for dancers to attend carefully to postures and movements. Further investigation is required to examine whether such attention prevents injury.

A major motivator for dancers to maintain unique postures and movements is the aesthetic demands of dance. Some dancers defined ideal posture as based on aesthetic standards, striving for an “unnatural perfection”. This implies dancers are willing to compromise what they consider to be optimal biomechanics for artistic purposes, exemplified by the increasing elevation angle of the lifting leg, which is argued to increase stress on adjacent regions and induce pain [37,38]. Thus, dancers express a quandary; to either attain specific postures to prevent injury, or exaggerate movement for aesthetics with possible impact on susceptibility to injury [37]. This duality was criticised by some dancers. To address this, some dancers expressed a need to seek and establish postures and movements that consider the uniqueness of their own body structure to achieve their perceived ideal posture as discussed below.

### 4.5. Strengths and limitations

The strength of this study is the inclusion of a large group of dancers from highly competitive elite dance companies and University programs. The findings of this study need to be considered in light of several limitations. First, only 22% of the participants identified as ballet dancers and views might be biased to those of contemporary dancers. Of relevance here, contemporary dance is not restricted by classical ballet conventions and can include more fluid movements [39]. As it was impossible identify the identity of all contributors in the transcriptions it is not possible to relate each comment to the dance training or experience of the individual responsible. Second, the study involved ballet and contemporary dancers from New York City, and findings may not be transferable to individuals in other environments where there might be different concepts of dance training, different cultures, and so on. Third, a weakness of focus group as a data collection method is the potential for dominance by outspoken individuals, as their opinions can potentially influence the other participants’ answers.

### 4.6. Practical and clinical applications and implications

Insights into dancers’ perspectives of posture and movement have potential relevance for conceptualisation and rehabilitation of posture and movement in non-dancers. Although our qualitative study cannot determine whether the perspectives of dancers are accurate, it does constitute the opinion of “experts” in posture and movement and justifies consideration of several issues.

First, the conceptualisation of dancers that ongoing conscious effort is required to fine tune the “quality” of their posture and movement and prevent deterioration of their desired form suggests that when a patient/client is asked to change their posture or movement, it might be unrealistic for clinicians to expect permanence and that ongoing maintenance might be required.

Second, considering the individual variation in anatomy, physiology, and biomechanics, rather than aiming for a single ideal, individualisation of training and individualisation of the goal is likely to be necessary.

Third, conceptualisation of posture as dynamic contrasts the common clinical focus on training individuals to maintain static postures [40]. This reinforces that posture involves a balance of movement and stiffness that depends on the demands of the task and are never completely static [5,41]. For function, training of posture is likely to benefit from be dynamic within diverse environments, with varying sensory feedback, and different levels of difficulty, in order to enhance posture’s adaptability and transferability.

Fourth, although this study does not resolve the debate whether posture and movement are relevant for prevention and management of pain and injury, it highlights potential explanations for the apparent contradiction between dancer’s high rates of pain and injury, despite enviable quality. Namely, the contrasting issues of optimisation of posture and movement to prevent injury, and risk derived from demand to achieve unnatural tasks.

## 5. Conclusion

This study explored dancers’ conceptualisations and beliefs about posture and movement. Results demonstrate that dancers have a detailed and multifaceted understanding of posture and movement. They reconcile the contradiction between good posture and movement and injury by the extreme demands required to achieve aesthetics of dance. The observations provide both insight into critical aspects of the perspective of dancers and implications for clinicians who train posture and movement and treat musculoskeletal conditions.

## Supporting information

S1 File Semi-Structured Focus Group Guide. (DOCX)

S2 File High-resolution version of all themes and the interconnections between them. (TIFF)

S3 File Representative quotes. (DOCX)

S4 File Inclusivity in global research questionnaire. (DOCX)
