# Supplementary material for: Professional dancers’ beliefs and conceptualisations of their posture and movement: A qualitative research study
Source: PLoS One. 2026 Feb 9;21(2):e0339568. doi: 10.1371/journal.pone.0339568 (PMC12885312; doi:10.1371/journal.pone.0339568)
Supplement: S1 File — (DOCX) [file pone.0339568.s001.docx]

**Supplement S1: Semi-Structured Focus Group Guide**

- *Note that questions will be kept very open with minimal prompts*

1. When I say posture, what does it mean to you? What, do you think about.
2. How often do you think about your posture/position/alignment
3. What triggers you to pay attention to your body posture/position/alignment
4. When you pay attention to your posture/position/alignment, what do you commonly change
5. Why do you change body posture/position/alignment, what benefit do you get?
6. What are the key features of the posture/alignment you work to achieve?
7. To what degree do you feel you can achieve the ideal. Is it difficult?
8. How much do you feel that it is built in/automatic?
9. Do you think that your dance training changed how you use your body in everyday life? How?
10. In an average day how often are you consciously aware of how you move?
11. When you pay attention to your movement, what do you try to achieve or what do you commonly change?
12. Is there anything else you would like to mention that we haven’t discussed today?
