# Supplementary figures and images for: Professional dancers’ beliefs and conceptualisations of their posture and movement: A qualitative research study
Source: PLoS One. 2026 Feb 9;21(2):e0339568. doi: 10.1371/journal.pone.0339568 (PMC12885312; doi:10.1371/journal.pone.0339568)

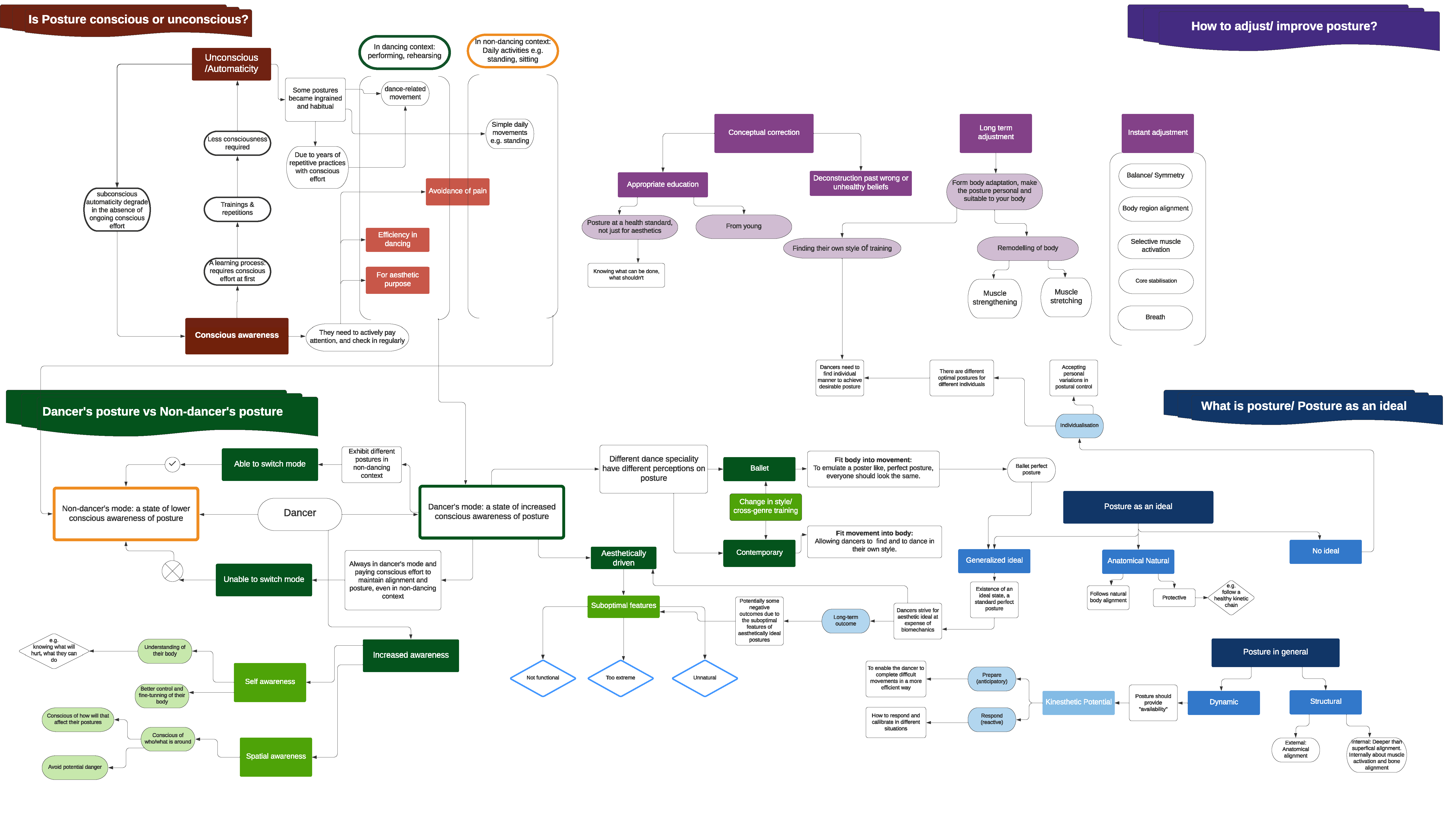

Supplement: S2 File — (TIFF) [file pone.0339568.s002.tiff]
