# Supplementary material for: Professional dancers’ beliefs and conceptualisations of their posture and movement: A qualitative research study
Source: PLoS One. 2026 Feb 9;21(2):e0339568. doi: 10.1371/journal.pone.0339568 (PMC12885312; doi:10.1371/journal.pone.0339568)
Supplement: S3 File — (DOCX) [file pone.0339568.s003.docx]

**Supporting information 3. Representative quotes.**

| **Theme 1: Posture being conscious or subconscious** | | |  |
| --- | --- | --- | --- |
| **Posture is controlled by conscious awareness** | **Avoidance of pain** | *“I do actively think of my posture, where my public alignment is, how I'm holding my, my shoulder is if I have a backpack on where I'm placing, stacking my bones as I physically climb the stairs to limit the pain or discomfort I might experience on my knees.”* | *T1Q1* |
|  | **Improvement of efficiency in dancing** | *“When I’m dancing I’m constantly checking in. Just because like, generally when I am unable to achieve a step it’s generally due to alignment. And so if I go back to center, realign, I'm able to execute it.”* | *TQ2* |
|  | **Aesthetic purposes** | *“So then I started thinking more and more of it, trying to get back the aesthetically good posture, ballet standard posture.”* | *TQ3* |
| **Posture can be subconscious** | **Training effect** | *“That it doesn't feel like I am doing it on purpose to your point of our body knows what to do. It's almost like it is a subconscious unconscious thing that just occurs because of the training. “* | *T1Q4* |
|  | **Translation to daily activities** | *“But it's just sort of it's becoming ingrained to us to have our shoulders back our chest be sort of up, be standing straighter than necessary”* | *T1Q5* |
| **Level of consciousness can alter and it is a reversible process** | **Translation of conscious to subconscious, after practices** | *“I know that I tend to climb stairs with my quads, rather than using my glutes. And so just making sure to, like, consciously do it. Now that I've sort of gotten more into the habit of it. And to think about it less, I can just check and be like, Oh, yeah, I'm doing it, as opposed to each step thinking about it.”* | *T1Q6* |
|  | **Loss of subconscious control** | *“So the ideal that you try to achieve doesn't necessarily become automatic, because if you don't practice it, you lose some of it.”* | *T1Q7* |
| **Theme 2: Dancer’s posture vs Non-dancer’s posture** | | |  |
| **Posture in dancer’s mode:** | *“When I'm in the dance environment I'm constantly aware of, you know, am I placing my feet, my knee tracking right over my feet? Is my spine aligned?”* | | *T2Q1* |
|  | **Suboptimal features at expense of biomechanics** | **Too extreme: “***There's that thing that happens in dance in general, where you can push too far from one and from, from one suggestion of what's perfect, way over to an extreme right and then puts sort of weird habits on the bone structure.”* | *T2Q2* |
|  |  | **Not functional:** “*Yes, but those are dance, that's not functional. We are asked to do crazy. It's all for, and what is it called? No and like aesthetics, it's all for aesthetics, you want to look cool.”* | *T2Q3* |
|  |  | **Unnatural:** *“It's not a natural way for our bodies to be enjoying it. The way we're saying it the way we do this. It's meant to be, you know, it's not human, it's like, really like force, like turning out.”* | *T2Q4* |
|  | **Dance style specificity** | **Contemporary***: “But in contemporary dance, because it's more movement based, it's more fluid that allows you to figure out how your body reacts to the movement. And like how you make it your own and how it works for you in the sense.”* | *T2Q5* |
|  |  | **Ballet:** *“But I’m gonna speak to ballet, like there is an ideal. And generally one of twenty dancers on stage where you all feel the same.”* | *T2Q6* |
| **Posture in non-dancer’s mode** | *“In my daily life, the only time I, I mean I try to prepare myself, but I don't really think about as much”* | | *T2Q7* |
|  | **Unable to switch mode** | *“So it's, it's sort of, I think, my, my thinking has shifted to needing to think of, think about my posture when I'm outside of dance, so that so that I don't develop bad habits that would impact dancing.”* | *T2Q8* |
|  | **Able to switch** | *“Yeah, I don't think that I'm actively aware of stacking those bones anymore. Just kind of switch into that mode.”* | *T2Q9* |
| **Theme 3: What is posture/posture as an ideal** | | |  |
| **Posture is structural:** | *“I think once we all figure out how our bodies anatomically align correctly. Then comes the posture.”* | | *T3Q1* |
|  | *“Posture being more superficial thing where, where the skeleton sort of sits and rests, and muscles and ligaments affect where the bones sit.”* | | *T3Q2* |
| **Posture is dynamic** | **Availability / Kinaesthetic potential** | **To prepare***: “Like enabling yourself to organize your body in such a way that you can achieve these tasks, optimising our bodies for these things, preparing all this stuff.”* | *T3Q3* |
|  |  | **To respond:** *“So the posture has to be this thing, that readily available, has availability to shift with a quick so it doesn't crack because it's brittle.”* | *T3Q4* |
|  |  | **Pendulum and baseline:** *“It’s kind of the base. And I can move from that base in either direction, not spatially direction forward or backwards. But it's like a pendulum, like posture is where there's the downswing, the ease in between can go to either spectrum from that neutral posture.”* | *T3Q5* |
| **Posture as an ideal** | **Generalised Ideal** | *“So then I started thinking more and more of it, trying to get back the aesthetically good posture, ballet standard posture.”* | *T3Q6* |
|  |  | **“***Perfect posture is not natural***”***.* | *T3Q7* |
|  |  | *“And then you take that on as perfect posture. So then you leave the room and attempt to hold yourself that we constantly which can become actually unhealthy.”* | *T3Q8* |
|  | **A natural, safe, and healthy state is ideal:** | **Natural***: “And like where it naturally wants to be plugged in. It's more, it's like a neutral, grounded place that the bone is, has evolved to be in a human body.”* | *T3Q9* |
|  |  | **Healthy***: “What is good just to be functioning at a normal, healthy kinetic chain.”* | *T3Q10* |
|  | **No ideal state** | **“***Just observing how different postures serve different purposes, and realising that there isn't one posture that is an ideal”* | *T3Q11* |
|  |  | **Individualised optimism:** *“I think it's like, perfect versus optimal. I feel like not going towards the perfect, but going towards the optimal for you. “* | *T3Q12* |
|  |  | **Realisation comes with maturity and experience**: “*And I think all of us probably went through a phase where we are also trying to get that and you get older, and then you gain a lot of information, you do other dance styles, and then you start to learn on your own how to take on a more intelligent way to move. And I think a lot of that just comes with like maturity of learning your body, and maybe not so much like, you know, harmful training, sometimes it can do that. But sometimes it's just, you just need the time to realize it.”* | *T3Q13* |
| **Theme 4: How to adjust or improve posture** | | |  |
| **Correction through instant adjustment:** | **Anatomical alignment** | *“I like to think about all the points I can make where, where do they fall on my body”.* | *T4Q1* |
|  | **Muscle activation** | *“I try to aspire stronger activation of the right hamstring, and stronger glutes. And so I can properly create, I could properly activate my hamstring to lengthen so I can put myself in a neutral position to lengthen through my lower lumbar back*.” | *T4Q2* |
|  | **Change position** | *“I've been in this same position for a while, I should like shift or I'm always like moving on even in this chair like notice I'm just like rocking back and forth.”* | *T4Q3* |
|  | **Core engagement** | *“How am I using my body to ensure I'm not hurting myself, so that idea of like, I'm going to lift something heavy, so and engage my core lift from the legs.”* | *T4Q4* |
|  | **Breathing** | *“And breathing helps integrate all of that stuff, right? It helps access my blood flow and helps your muscles move.”* | *T4Q5* |
| **Correction over long term** | *“And I think this part takes time, internally, like strengthen deep muscles that support that structure, and make it more personal.” Another response is to train in their own style. what's helped me support that is actually my movement training, whether Pilates, weightlifting, or, that's helped me. But if I just kept going one cell to the next, I'm like getting lost in translation, my body just gets boxed up too much sugar gets too much there, I need to have my own practice in between to support myself.”* | | *T4Q6* |
|  | *“So I feel like even across the other side of the dance, you have to adjust your posture, sort of like deconstruct what you think posture is.”* | | *T4Q7* |
| **Conceptual correction** | *“I think that generally in the dance world, I think, in education there, there can be more, you know, with anatomy and lessons and while there while the dancers are still young.”* | | *T4Q8* |
